# Development and validation of a single latent variable self-reported periodontal disease scale based on the disease’s common signs and symptoms in Saudi adults

**DOI:** 10.1186/s12903-025-05804-x

**Published:** 2025-03-23

**Authors:** Yasmine N. Alawaji, Mohamed H. Alqasoumi, Saleh N. Alwatban, Abdulaziz M. Halwani, Lamya A. Aljnoubi, Bayan K. Alshehri, May K. Alenezi

**Affiliations:** 1 Preventive Dental Science Department, College of Dentistry, King Saud bin Abdulaziz University for Health Sciences, Riyadh, Saudi Arabia; 2 King Abdullah International Medical Research Center, National Guard Health Affairs, Riyadh, Saudi Arabia; 3 College of Dentistry, Majmaah University, Majmaah, Saudi Arabia; 4 College of Dentistry, Vision Colleges, Riyadh, Saudi Arabia

**Keywords:** Self-reported periodontal disease, Validation, Scale

## Abstract

**Background:**

Many studies attempted to evaluate and improve the accuracy of self-reported periodontal disease questionnaire to provide a feasible tool for screening the disease. The aim of this study was to develop and validate a self-reported periodontal disease screening scale (SRPDSS) in Saudi adults and to identify the association of periodontal disease with sociodemographic determinants and oral health behaviors.

**Methods:**

The data was collected digitally from Saudi adults (≥ 18 years) in Riyadh city using a questionnaire that inquired about sociodemographic characteristics, oral health behaviors and self-reported periodontal disease. The SRPDSS was developed using items from the literature or suggested by this study authors. The scale was validated for its construct validity and psychometric properties.

**Results:**

A total of 559 participants met the eligibility criteria out of 894 invited individuals. The mean (SD) age was 31.7 (12.7) and 68.5% of the participants were women. The scale items were selected based on common signs and symptoms of periodontal disease and the scale was confirmed to have a total of 10 items and a single latent variable using confirmatory factor analysis. The internal consistency using Cronbach alpha was acceptable = 0.75 and the test re-test reliability using Spearman’s correlation coefficient was excellent = 0.93. The final scale’s goodness of fit was acceptable as indicated by the Root Mean Square Error of Approximation (RMSEA) = 0.078, upper bound of the RMSEA 90% CI = 0.093, and the Standardized Root Mean Square (SRMR) = 0.059. Using linear regression analysis, the self-reported periodontal disease had statistically significant associations with age > 30 years; coefficient: 1.19 (95% confidence interval [CI]: 0.22, 2.70), lower parents’ income; coefficient: 1.5, (95% CI: 0.58, 2.42), lower parents’ education; coefficient: 1.1, (95% CI: 0.28. 1.92), regular dental visits; coefficient: -1.79 (95% CI: -2.70, -0.89), regular toothbrushing; coefficient: -1.51 (95% CI: -2.32, -0.70).

**Conclusions:**

This study developed and validated a 10-item self-reported periodontal disease screening tool based on its signs and symptoms in Saudi adults. The self-reported periodontal disease was significantly associated with older age, lower parents’ socioeconomic status, irregular brushing, and lack of regular dental visits.

**Supplementary Information:**

The online version contains supplementary material available at 10.1186/s12903-025-05804-x.

## Background

Periodontal disease is a group of diseases of that includes gingivitis, which is a reversible inflammation of gingival tissues, and periodontitis, an irreversible destruction of the periodontal tissues [1]. If periodontitis is left untreated, it may progress to tooth loss, loss of masticatory function, and compromised systematic health and quality of life [2–5]. The estimation of the prevalence of periodontal disease and its associated determinants in the population is important to plan and evaluate the effectiveness of preventive programs [6–8]. In 1992, the periodontitis prevalence in Saudi adult population ranged from 21 to 32% in the central region of Saudi Arabia [9]. Other studies estimated the prevalence of periodontal diseases in non-representative samples of the Saudi population recruited from hospitals, dental school clinics, and schoolchildren which ranged from 46.6 to 100.0% for gingivitis and 3.2 to 85.4% for periodontitis [10–15].

A comprehensive periodontal examination can take up to 40 min to record the full mouth probing depths and clinical attachment loss per individual [16]. The application of such periodontal examination in nationally representative surveys can be time exhaustive, costly, and require several examiners [6]. The use of periodontal indices, partial mouth recording protocols, and self-reported periodontal disease have been suggested as approaches to overcome the inherent challenges to conduct national surveys in representative samples [6, 17–19]. Though, these alternative approaches have their own limitations and several studies have been testing and developing the methods for improving their validity and accuracy [17, 18, 20–25].

A self-reported periodontal disease oral health questionnaire has been developed by the U.S. Centers for Disease Control and Prevention and the American Academy of Periodontology (CDC/AAP) to offer a more time-efficient screening tool [26]. This self-reported questionnaire is intended to be used to evaluate the time trend prevalence of periodontitis in the U.S [27]. The scale by the CDC/AAP demonstrated a wide range of diagnostic accuracy when studied in several populations [24, 26, 28–30]. When analyzed separately, the pooled sensitivity of each of the CDC/AAP self-reported periodontal disease items was poor and ranged from 15.9% to 54.9% for moderate and severe periodontitis while the pooled specificity had high diagnostic accuracy ranging from 79.5% to 94.7% for moderate and severe periodontitis [19]. In recent studies, the area under the receiver operating characteristic curve (AUC) had an acceptable discrimination ≥ 0.7 for severe periodontitis and a low discrimination for total periodontitis [22, 25, 30].

Several studies combined determinants such as age, sex, socioeconomic status, oral self-care, and smoking status with the self-reported periodontal disease items and used the total score to improve the diagnostic accuracy of self-reported periodontal disease [24–26, 28, 30]. However, including these determinants within the summarized score of self-reported periodontal disease my preclude its use for studying the exposures associated with the periodontal disease in populations. In addition, majority of the previous studies focused on the assessment of diagnostic accuracy separately for each scale item; such approach might be limited since each item may be restricted in its ability to identify the overall periodontal diagnosis [19, 24, 26]. Alternatively, the use psychometric theory can provide a useful summary of periodontal disease based on its overall inherent characteristics [31]. Recently, a multidimensional self-reported scale has been developed and validated using psychometric theory which had two latent variables and a total of 17 items [23]. The use of multiple dimensions can be complex in interpreting and summarizing the scale’s score, it is thus preferable to have a single latent variable. Therefore, our study aims to develop and validate the presence of a single latent variable self-reported periodontal disease screening scale (SRPDSS) based on its signs and symptoms. A secondary objective was to test the developed scale’s ability to identify the sociodemographic determinants and oral health behaviors associated with the self-reported periodontal disease.

## Methods

### Study population

**Fig. 1:**
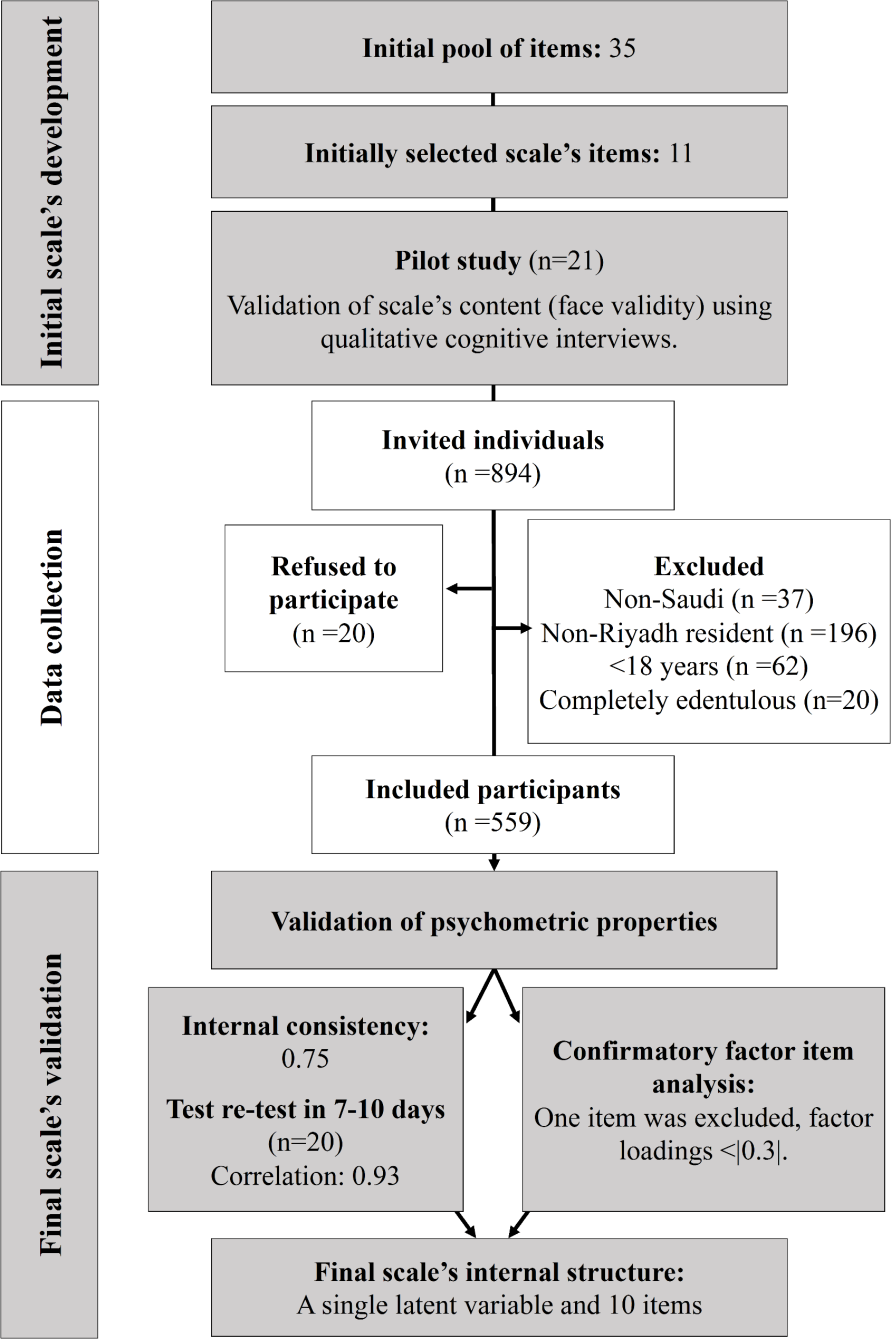
Flowchart of the study design

Eligibility for inclusion in the study was limited to Saudi adults who are ≥18 years and residing in Riyadh city. Individuals reported to have lost all their teeth were excluded **(**Fig. 1**)**. Institutional Review Board (IRB) ethics approval for the study was obtained from King Abdullah International Medical Research Center (**RYD-23-419812-116041**) and consent forms were obtained prior to data collection. The data were collected between August to December 2023.

### Data collection

Due to lack of a sampling frame and for feasibility reasons, a nonprobability sampling (combination of convenience and snowballing techniques) was utilized to recruit the study’s population; study’s population were invited and recruited from social media platforms, shopping malls, or referrals from previously included individuals [32, 33]. The questionnaire was distributed digitally and inquired about sociodemographic characteristics, oral health behaviors, medical conditions, and total number of missing teeth. In addition, periodontal disease was assessed utilizing the SRPDSS.

### Statistical analyses

All statistical analyses were conducted using STATA software, version 17.0 (TX, USA). The estimated minimum sample size was 110 individuals based on requiring a 10 individuals per scale’s item for conducting a confirmatory factor analysis [31]. The background characteristics were described using univariate descriptive analysis. For identifying the determinants associated with the periodontal disease, a simple linear regression analysis was conducted and a p-value < 0.05 was considered significant. The dependent variable was the summed score of the self-reported periodontal disease. The independent variables were age in years converted into binary outcome: ≤30 years (reference) and > 30 years, sex (binary): male (reference) and female, parents’ education level converted into binary outcome: > High school (reference) and ≤ high school, parents’ income in Saudi Riyal (SAR) converted into binary outcome: ≥10,000 SAR (reference) and < 10,000 SAR, regular dental visits (binary): no (reference) and yes, regular toothbrushing converted into binary outcome: no (reference) and yes, and smoking status converted into binary outcome: non-smokers (reference) and smokers.

### The SRPDSS development and validation process

#### Conceptual item analyses

To develop the periodontal disease self-reported scale, a deductive approach was followed and a total of 35 pool of items was collected from several sources listed in the supplementary table (Table S1) [31]. The initial pool of items had a total of 8 scale items suggested by the CDC/AAP [19, 24], 4 items listed in a study by Carra *et al.* [28], 22 items listed in a study by Wright *et al.* [23], and a new item was added by the authors of this study “*have you been diagnosed with deep gum inflammation by your dentist*?”. The pool of items was evaluated conceptually based on the periodontal disease signs and symptoms; items were eliminated if had any of the following weaknesses: irrelevant to periodontal disease signs and symptoms, non-specific to periodontitis, redundant, or uncommonly reported by periodontitis patients [31]. Accordingly, a total of 24 items were eliminated and the reasons behind exclusion were presented in Table S1. For example, two of the excluded CDC/AAP scale items asked about the oral self-care (use of mouthwash and interdental aids) because they do not directly measure the periodontal disease signs and symptoms. Thus, a hypothesized scale’s internal structure had a single latent variable for self-reported periodontal disease and a total of 11 selected items for the SRPDSS.

#### Response formats

The response formats were made as following: a 3-point response: yes, no, and I don’t know for questions about gum inflammation, deep gum inflammation, deep gum cleaning, gum recession, bone loss, and tooth appearance [26]. The rest of the questions had a 5-point response format ranging from never to always for questions about gum tenderness, gum bleeding, tooth mobility, and chewing mobility. A 5-point response format ranging from very poor to excellent for questions about rating the overall teeth and gum health. The questionnaire and its responses were translated from English into Arabic language using forward backward method.

#### Pilot study

A pilot study was done (n = 21 individuals) to confirm the scale’s face validity using the Arabic version of the scale [31]. The pilot study was conducted to ensure the clarity of the questionnaire, proper understanding by the non-professionals, evaluation of the questionnaire presentation and its response formats, and to estimate the answering time as perceived by the participants. Three qualitative cognitive interview methods were utilized: think aloud (sharing the thinking process), rephrasing, and probing (asking follow-up questions to achieve an in-depth assessment) [31]. The bone loss question *“Have you ever been told by a dental professional that you lost bone around your teeth?”* was found confusing by participants as they thought this could be related to bone loss after tooth extractions. The question was modified to make it more specific as following: *“Have you ever been told by a dental professional that you lost bone around your teeth (excluding bone loss due to tooth extraction)?”.* Another question which needed more clarification was *“Have you ever had any teeth become loose on their own*,* without an injury?”* one of the participants asked if this includes movement during orthodontic treatment. Accordingly, the response format was changed from (yes, no, I don’t know) into a 5-point response ranging from never to always where often to always can be more relevant to periodontitis rather than occasional or short-term increase in tooth mobility. In addition, the question *“Do you have any missing permanent teeth?”* was found confusing if it should include extracted teeth after their replacement. Thus, the question was modified to *“Do you have any missing permanent teeth aside from replacement with artificial teeth?”.* The recruitment in the pilot study stopped when the participants no longer had any confusion or suggestions to improve the questionnaire.

#### Psychometric analyses

For psychometric analyses, the hypothesized internal structure of the SRPDSS containing a single latent variable and 11 items was examined using confirmatory factor item analysis [31]. The 3-point response formats of the SRPDSS items were re-coded, such that *“No”* and “*I do not know”* were combined. Thus, the response format became binary: “yes” and “no”. The 5-point response formats were not modified. Maximum likelihood was used as an estimation approach since the listwise missingness was minimum (< 5.0%). The factor loadings were considered unacceptable if <|0.3|. Goodness of fit indices indicate adequate fit if Root Mean Square Error of Approximation (RMSEA) was < 0.08, upper bound of 90.0% confidence interval (CI) for the RMSEA was < 0.10, Standardized Root Mean Square (SRMS) < 0.08, and Comparative Fit Index (CFI) was ≥ 95 [31, 34–36]. Modification indices were used to improve the goodness of fit if it did not meet the criteria. For the test re-test stability, duplicate responses from a subsample of 20 individuals were collected 7–10 days apart and it had an excellent correlation = 0.93 using Pearson’s correlation coefficient [31, 37]. The internal consistency of the SRPDSS was tested using Cronbach alpha.

## Results

Among 894 invited individuals to participate in the study, a total of 559 participants met the eligibility criteria and 20 individuals refused to participate (response rate was 97.8%). A total of 315 individuals were excluded due to being non-Saudis, non-Riyadh residents, age < 18 years, or completely edentulous as depicted in the flowchart (Fig. 1). The second responses of the individuals who completed the questionnaire twice for test-retest stability were included. The background characteristics are listed in Table 1 where the mean (SD) age was 31.8 (12.7) and 68.5% of the participants were women. The distribution of responses to each of the SRPDSS items were outlined in Table 2. For two items that asked about periodontitis signs, based on their diagnoses by their dentists: *“bone loss”* and “*deep gum inflammation*”, 6.2% and 13.5% answered “*yes”* respectively.

**Table 1:** Distribution of background characteristics of participants included in the study

Determinants		Distribution
Age in years, mean (SD)		31.8 (12.7)
Sex, n (%)	Male	176 (31.5)
	Female	383 (68.5)
Marital status, n (%)	Single	324 (58.9)
	Married	201 (36.6)
	Divorced	17 (3.1)
	Widowed	8 (1.5)
Parents’ education Level, n (%)	Primary school	35 (6.8)
	Elementary school	38 (7.4)
	Secondary school	153 (29.6)
	Diploma	50 (9.7)
	Bachelor	83 (16.1)
	Master’s	55 (10.6)
	Doctoral	64 (12.4)
Parents’ income in Saudi Riyal (SAR)	Lower (< 10,000 SAR)	258 (63.4)
	Higher (≥ 10,000 SAR)	149 (36.6)
Smoking status, n (%)	Non-smokers	432 (85.0)
	Smokers	76 (15.0)
Regular dental visits, n (%)	Yes	166 (32.3)
	No	348 (67.7)
Regular tooth brushing (twice a day), n (%)	Yes	258 (50.3)
	No	255 (49.7)
Use of interdental aid, n (%)	Yes	257 (51.3)
	No	244 (48.7)
Missing teeth, mean (SD)		3.4 (0.5)

**Table 2:** The self-reported periodontal disease screening scale’s (SRPDSS) pool of items and their distributions in the study’s population

SRPDSS items	Frequency: *n* (%)	
1. Gum disease Do you think you have gum disease?	Yes No I don’t know	120 (24.3) 250 (51.8) 120 (23.6)
2. Deep gum inflammation Have you been diagnosed with deep gum inflammation by a dentist?	Yes No I don’t know	69 (13.8) 385 (76.7) 48 (9.6)
3. Deep cleaning Have you ever received a deep cleaning for treatment of your gums?	Yes No I don’t know	101 (20.3) 364 (73.2) 32 (6.4)
4. Bone loss Has your dentist ever told you that you have lost bone around your teeth (excluding extracted teeth)?	Yes No I don’t know	30 (6.2) 428 (86.7) 35 (7.1)
5. Gum recession Do you have receding gums or do your teeth look longer than they used to?	Yes No I don’t know	78 (16.3) 316 (65.8) 86 (17.9)
6. Tooth appearance. During the past three months, have you noticed that one of your teeth looks different?	Yes No I don’t know	115 (23.7) 329 (67.8) 41 (8.5)
7. Tooth mobility Have your teeth ever moved on their own without injury?	Never Rarely Often times Very often Always	310 (62.9) 104 (21.2) 57 (11.6) 19 (3.9) 3 (0.6)
8. Mobility on chewing Do you feel that your teeth move while chewing food?	Never Rarely Often times Very often Always	386 (81.8) 47 (10.0) 28 (5.9) 9 (1.9) 2 (0.4)
9. Gum bleeding During the past three months, have you noticed bleeding gums?	Never Rarely Often times Very often Always	193 (40.4) 117 (24.5) 123 (25.7) 27 (5.7) 18 (3.8)
10. Gum tenderness Do you feel discomfort in your gums?	Never Rarely Often times Very often Always	234 (49.4) 104 (21.9) 89 (18.8) 28 (5.9) 19 (4.0)
11. Overall dental and gum health In general, how do you rate the health of your teeth and gums?	Excellent Very good Good Poor Very poor	80 (16.10) 199 (40.0) 131 (26.4) 65 (13.1) 22 (4.4)

**Fig. 2:**
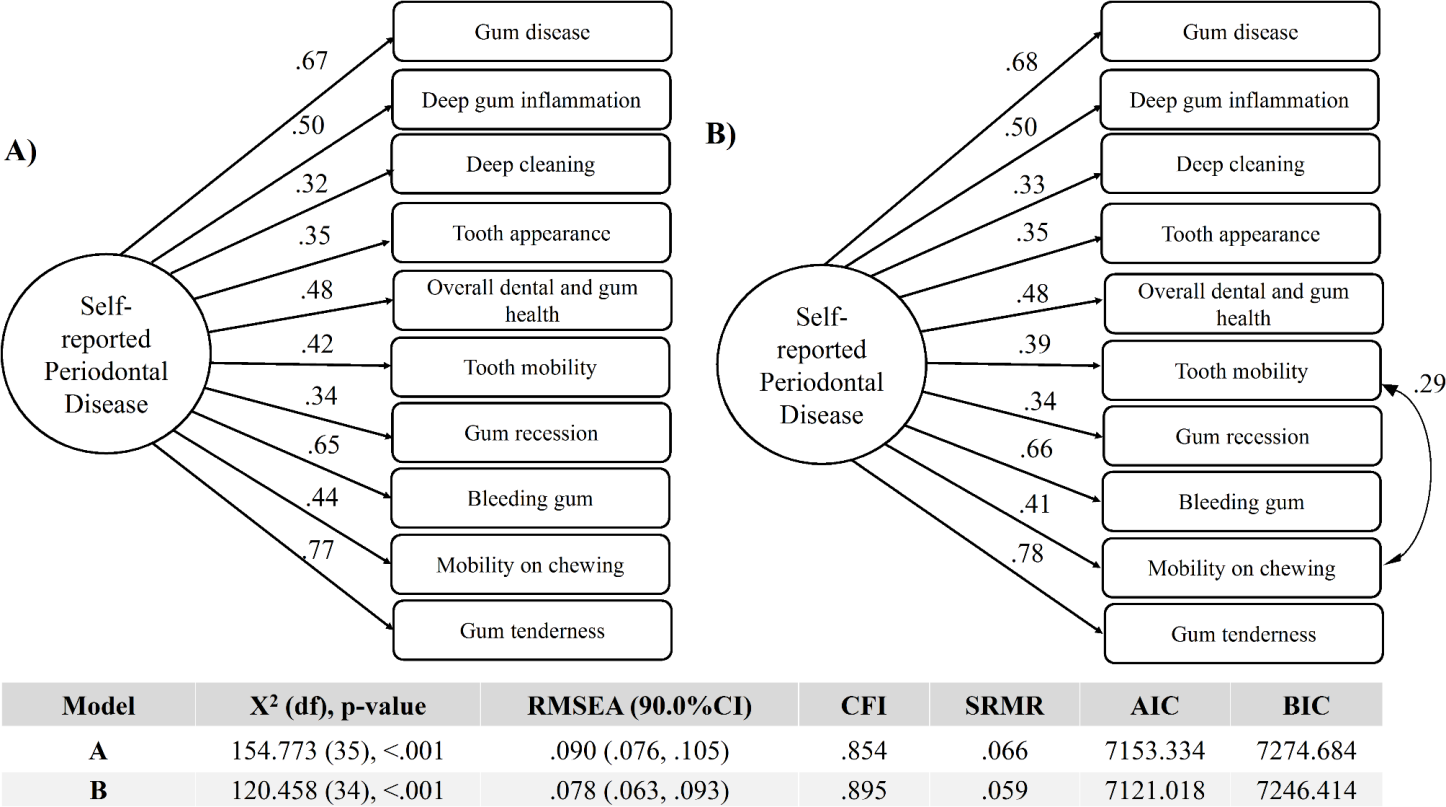
The self-reported periodontal disease screening scale internal structure has one latent variable and 10 items using a confirmatory factor item analysis. **(A)** The model had an unacceptable goodness of fit based on the Chi-square (X^2^) p-value < 0.001, Root Mean Square Error of Approximation (RMSEA) > 0.08 and the upper bound of RMSEA 90% confidence interval (CI) > 0.10, Comparative Fit Index (CFI) < 0.95. **(B)** Modified model with correlation between “tooth mobility” and “mobility on chewing” items had acceptable goodness of fit as indicated by the RMSEA < 0.08, upper bound of RMSEA 90% CI was < 0.10, and Standardized Root Mean Square (SRMR) < 0.08

Based on the confirmatory factor item analysis, one item had poor factor loading (<|0.3|) and was eliminated: *“bone loss”.* The SRPDSS’ internal structure has been confirmed to have a single latent variable and a total of 10 items (Fig. 2). The initial model did not have adequate goodness of fit as indicated by the Chi-square p-value < 0.001, SRMR > 0.08 and the upper bound of the RMSEA 90% CI > 0.10, and the CFI < 0.95 [31, 34–36]. The modified model with added correlation between “*tooth mobility*” and “*mobility on chewing”* items had acceptable goodness of fit as indicated by the RMSEA < 0.08, upper bound of the RMSEA 90% CI was < 0.10, and the SRMR < 0.08. The internal consistency of the SRPDSS was tested using Cronbach alpha and had an acceptable score = 0.75.

The self-reported periodontal disease had statistically significant associations (Table 3) with age > 30 years coefficient: 1.19 (95% confidence interval [CI]: 0.22, 2.70), lower parents’ income (< 10,000 SAR), coefficient: 1.5, (95% CI: 0.58, 2.42), lower parents’ education (< high school), coefficient: 1.1, (95% CI: 0.28. 1.92), regular dental visits, coefficient: -1.79 (95% CI: -2.70, -0.89), and regular toothbrushing, coefficient: -1.51 (95% CI: -2.32, -0.70).

**Table 3:** The self-reported periodontal disease associated determinants in Saudi adults in Riyadh City using univariate linear regression analysis

Determinant		Coefficient (Slope)	*P*-value	95% CI	Intercept (Adjusted *R*^2^)
Age	≤ 30 years *reference				
	> 30 years	**1.19**	**0.017**	**(0.22**,** 2.70)**	**1.03%**
Sex	Male *reference				
	Female	0.48	0.363	(-0.56, 1.51)	-
Parents’ education level	> high school *reference				
	≤ High school	**1.10**	**0.009**	**(0.28**,** 1.92)**	**1.13%**
Parents’ income	Higher (≥ 10,000 SAR) *reference				
	Lower (< 10,000 SAR)	**1.50**	**0.002**	**(0.58**,** 2.42)**	**2.22%**
Regular dental visits	No *reference				
	Yes	**-1.79**	**< 0.001**	**(-2.70**,** -0.89)**	**2.68%**
Regular toothbrushing	No *reference				
	Yes	**-1.51**	**< 0.001**	**(-2.32**,** -0.70)**	**2.39%**
Smoking status	Non-smokers *reference				
	Smokers	-0.22	0.697	(-1.31, 0.88)	-

CI: Confidence interval, SAR: Saudi Riyal

## Discussion

Our study developed, validated and confirmed the reliability of the self-reported periodontal disease screening tool for Saudi adults in Riyadh. The scale’s internal structure was confirmed to have 10-items and a single latent variable. The self-reported periodontal disease was significantly associated with age older than 30 years, lower parent’s income and education level, irregular toothbrushing, and lack of regular dental visits.

Majority of previous studies focused on validating the self-reported periodontal disease questionnaires for their diagnostic accuracy and recommended variety of approaches for using few scale items in addition to background characteristics to achieve an acceptable diagnostic accuracy [24–26, 28, 30]. These studies did not evaluate the scale’s psychometric properties including the scale’s internal structure. In a study by Wright *et al.* a multidimensional scale was developed and validated with a total of 17-items [23]. The scale’s internal structure was initially tested using an exploratory factor analysis then confirmed the structure using a confirmatory factor analysis. Such approach can be limited due to the potential subjectivity and lack of stability of suggested solutions obtained using the exploratory factor analysis [38]. In contrast, our study used the confirmatory factor analysis first to evaluate the initially hypothesized structure based on conceptual item analysis of periodontal disease signs and symptoms during which we eliminated any redundant, uncommon, or irrelevant items [31]. The resulting unidimensional scale and 10-items in our study have an added advantage of simplicity in using and summarizing the scale scores rather than having a multidimensional scale.

One item was eliminated: “*bone loss*” due to poor factor loading. The “*bone loss*” was reported by a small proportion of study population (6.2%). In contrast, “*bone loss*” was reported by 14.4% of the study population a study by Carra *et al.* [28]. This difference in distribution might be due to the modification we made to the question to ask specifically about bone loss around teeth excluding extracted teeth. Despite that the three items “*bone loss*”, “*deep gum inflammation*” and “*deep cleaning*” were specific indicators of periodontitis, the latter two items were reported more frequently in our study population (13.5% and 20% of our study population, respectively). These differences in the frequency may reflect the differences in the terminologies communicated to the patients by their dental professionals in Riyadh, Saudi Arabia where the term “*bone loss*” may not be frequently used.

The primary objectives of epidemiological surveys are to describe the distribution of the diseases and the exposures associated with the disease within populations [39]. A commonly used method in previous studies is including exposures such as age, sex, smoking in the definition of the self-reported periodontal disease to obtain acceptable diagnostic accuracy [24, 25, 28–30]. Such approach could limit the use of self-reported periodontal disease measure in studying the exposures associated with the disease. In contrast, our study tested the developed scale’s function in studying the determinants associated with periodontal disease to fulfil one of the epidemiological surveys’ main objectives. The self-reported periodontal disease in the current study had significant associations with older age, lower parents’ socioeconomic status, irregular toothbrushing, and lack of regular dental visits consistent with known determinants of periodontal disease in the literature [8, 29, 40–42].

The strengths of our study include considering different approaches for validation such as cognitive interviews for construct validity, conceptual item analysis, and validation of psychometric properties including internal consistency, test-retest stability, and validation of internal structure using confirmatory factor analysis. The study has used a simple scale structure that retained the required information which is a desired endpoint of scale development. The limitations of our study include the inherent problems to the use of self-reported questionnaire including the potential limited understanding of the participant, and the recall bias. In addition, the participant’s education level and past dental history may influence the level of accuracy of the reported results. The study population was limited to Saudi adults living in Riyadh city which may limit the generalizability of the findings. Furthermore, the use of non-probability sampling may have introduced a selection bias which can limit the representativeness of our study population. Diagnostic accuracy has not been determined, and it could be examined in a future study where the SRPDSS scores could be compared with the different clinical diagnoses of periodontal diseases.

The theoretical implication of our study includes confirmation of the internal scale’s structure to have a single latent variable; thus, the scale’s score can be summed up or averaged to summarize the periodontal disease in populations. The scale also was confirmed to be able to study the determinants with periodontal disease in population. The practical implication of the study includes the possible use of the SRPDSS as a feasible and validated tool for screening the periodontal disease in Saudi national surveys. The internal consistency and test-retest stability were high which may support the potential use of the scale to evaluate the time trend prevalence of periodontal disease in the population.

## Conclusions

Our study developed and validated a single latent variable scale with 10-items for self-reported periodontal disease for its use in Saudi adult population. The self-reported periodontal disease was associated with older age, lower parents’ education level and income, irregular toothbrushing, and lack of regular dental visits. We recommend the use of self-reported periodontal disease scale as a feasible tool to study the disease distribution and its associated determinants. For future studies, we recommend testing the diagnostic accuracy of the study for further validation of the scale’s ability to screen periodontitis in the population.

## Electronic supplementary material

Below is the link to the electronic supplementary material.

Supplementary Material 1

## Data Availability

Data will be available upon request from the corresponding author.

## Abbreviations

SRPDSS: Self-reported periodontal disease screening scale
SD: Standard deviation
RMSEA: Root Mean Square Error of Approximation
SRMR: Standardized Root Mean Square
CFI: Comparative Fit Index
CI: confidence interval
CDC/AAP: The U.S. Centers for Disease Control and Prevention and the American Academy of Periodontology
AUC: Area under the receiver operating characteristic curve
IRB: Institutional review board
SAR: Saudi Riyal
X^2^: Chi square
df: Degree of freedom
