# Correction: Development and validation of a single latent variable self-reported periodontal disease scale based on the disease’s common signs and symptoms in Saudi adults

**DOI:** 10.1186/s12903-025-06102-2

**Published:** 2025-05-24

**Authors:** Yasmine N. Alawaji, Mohamed H. Alqasoumi, Saleh N. Alwatban, Abdulaziz M. Halwani, Lamya A. Aljnoubi, Bayan K. Alshehri, May K. Alenezi

**Affiliations:** 1 Preventive Dental Science Department, College of Dentistry, King Saud Bin Abdulaziz University for Health Sciences, Riyadh, Saudi Arabia; 2 King Abdullah International Medical Research Center, National Guard Health Affairs, Riyadh, Saudi Arabia; 3 College of Dentistry, Majmaah University, Majmaah, Saudi Arabia; 4 College of Dentistry, Vision Colleges, Riyadh, Saudi Arabia

**Correction** **:** **BMC Oral Health 25, 424 (2025)**

**https://doi.org/10.1186/s12903-025-05804-x**

In this article [1], the author would like to remove all the Arabic content from Table 2 which looks like symbols and not legible. The incorrect and correct version of Table 2 are given below.

The original article has been corrected.

Incorrect Table 2:

**Table 2:** The self-reported periodontal disease screening scale’s (SRPDSS) pool of items and their distributions in the study’s population

**SRPDSS items**	**Frequency: *n* (%)**	
1. Gum disease	Yes	120 (24.3)
Do you think you have gum disease?	No	250 (51.8)
؟ةثللا يف باهتلاب باصم كنأ دقتعت له	I don’t know	120 (23.6)
2. Deep gum inflammation	Yes	69 (13.8)
Have you been diagnosed with deep gum inflammation by a dentist?	No	385 (76.7)
؟نانسأ بيبط لبق نم ةثللا يف قيمع باهلإب كصيخشت مت له	I don’t know	48 (9.6)
3. Deep cleaning	Yes	101 (20.3)
Have you ever received a deep cleaning for treatment of your gums?	No	364 (73.2)
؟جالعلا فدهب قيمع فيظنت تيقلت نا قبس له	I don’t know	32 (6.4)
4. Bone loss	Yes	30 (6.2)
Has your dentist ever told you that you have lost bone around your teeth (excluding extracted teeth)?	No	428 (86.7)
؟(علخلا تالاح ءانثتساب) كنانسأ لوح ماظعلا تدقف كنأ نانسألا بيبط كربخأ نأ قبس له	I don’t know	35 (7.1)
5. Gum recession	Yes	78 (16.3)
Do you have receding gums or do your teeth look longer than they used to?	No	316 (65.8)
؟قباسلا يف هيلع تناك امم لوطأ ودبت كنانسأ وأ ةثللا يف راسحنا كيدل له	I don’t know	86 (17.9)
6. Tooth appearance.	Yes	115 (23.7)
During the past three months, have you noticed that one of your teeth looks different?	No	329 (67.8)
؟فلتخم ودبي كنانسأ دحأ نأ تظحال له ،ةيضاملا ةثالثلا رهشألا لالخ	I don’t know	41 (8.5)
7. Tooth mobility	Never	310 (62.9)
Have your teeth ever moved on their own without injury?	Rarely	104 (21.2)
؟ةباصإ نود اهسفن ءاقلت نم كنانسأ تكرحت نأ قبس له	Often times	57 (11.6)
	Very often	19 (3.9)
	Always	3 (0.6)
8. Mobility on chewing	Never	386 (81.8)
Do you feel that your teeth move while chewing food?	Rarely	47 (10.0)
؟ماعطلا غضم ءانثا كرحتت كنانسأ نأب رعشت له	Often times	28 (5.9)
	Very often	9 (1.9)
	Always	2 (0.4)
9. Gum bleeding	Never	193 (40.4)
During the past three months, have you noticed bleeding gums?	Rarely	117 (24.5)
؟ةثللا يف فيزن تظحال له ،ةيضاملا ةثالثلا رهشألا لالخ	Often times	123 (25.7)
	Very often	27 (5.7)
	Always	18 (3.8)
10. Gum tenderness	Never	234 (49.4)
Do you feel discomfort in your gums?	Rarely	104 (21.9)
؟ةثللا يف حايترا مدعب رعشت له	Often times	89 (18.8)
	Very often	28 (5.9)
	Always	19 (4.0)
11. Overall dental and gum health	Excellent	80 (16.10)
In general, how do you rate the health of your teeth and gums?	Very good	199 (40.0)
؟كتثلو كنانسأ ةحص ميقت فيك ،ماع لكشب	Good	131 (26.4)
	Poor	65 (13.1)
	Very poor	22 (4.4)

Correct Table 2:

**Table 2:** The self-reported periodontal disease screening scale’s (SRPDSS) pool of items and their distributions in the study’s population

**SRPDSS items**	**Frequency: *n* (%)**	
1. Gum disease	Yes	120 (24.3)
Do you think you have gum disease?	No	250 (51.8)
	I don’t know	120 (23.6)
2. Deep gum inflammation	Yes	69 (13.8)
Have you been diagnosed with deep gum inflammation by a dentist?	No	385 (76.7)
	I don’t know	48 (9.6)
3. Deep cleaning	Yes	101 (20.3)
Have you ever received a deep cleaning for treatment of your gums?	No	364 (73.2)
	I don’t know	32 (6.4)
4. Bone loss	Yes	30 (6.2)
Has your dentist ever told you that you have lost bone around your teeth (excluding extracted teeth)?	No	428 (86.7)
	I don’t know	35 (7.1)
5. Gum recession	Yes	78 (16.3)
Do you have receding gums or do your teeth look longer than they used to?	No	316 (65.8)
	I don’t know	86 (17.9)
6. Tooth appearance.	Yes	115 (23.7)
During the past three months, have you noticed that one of your teeth looks different?	No	329 (67.8)
	I don’t know	41 (8.5)
7. Tooth mobility	Never	310 (62.9)
Have your teeth ever moved on their own without injury?	Rarely	104 (21.2)
	Often times	57 (11.6)
	Very often	19 (3.9)
	Always	3 (0.6)
8. Mobility on chewing	Never	386 (81.8)
Do you feel that your teeth move while chewing food?	Rarely	47 (10.0)
	Often times	28 (5.9)
	Very often	9 (1.9)
	Always	2 (0.4)
9. Gum bleeding	Never	193 (40.4)
During the past three months, have you noticed bleeding gums?	Rarely	117 (24.5)
	Often times	123 (25.7)
	Very often	27 (5.7)
	Always	18 (3.8)
10. Gum tenderness	Never	234 (49.4)
Do you feel discomfort in your gums?	Rarely	104 (21.9)
	Often times	89 (18.8)
	Very often	28 (5.9)
	Always	19 (4.0)
11. Overall dental and gum health	Excellent	80 (16.10)
In general, how do you rate the health of your teeth and gums?	Very good	199 (40.0)
	Good	131 (26.4)
	Poor	65 (13.1)
	Very poor	22 (4.4)
