# Supplementary material for: Development and validation of a single latent variable self-reported periodontal disease scale based on the disease’s common signs and symptoms in Saudi adults
Source: BMC Oral Health. 2025 Mar 23;25:424. doi: 10.1186/s12903-025-05804-x (PMC11931856; doi:10.1186/s12903-025-05804-x)
Supplement: Supplementary file 1 — Supplementary Material 1 [file 12903_2025_5804_MOESM1_ESM.docx]

Supplementary document

**Table S4.** Pool of items from the literature and selection of the self-reported periodontal disease scale items

| Source | Specific objective | Pool of Items | Selection of scale items |
| --- | --- | --- | --- |
| The Centers for Disease Control and Prevention and American Academy of Periodontology  (Taylor et al., 2007) | Gum disease | 1. Do you think you might have gums disease? | Included |
|  | Overall dental and gum health | 1. Overall, how would you rate the health of your teeth and gums? | Included |
|  | Deep cleaning | 1. Have you ever had treatment for gum disease such as scaling and root planning, sometimes called “deep” cleaning? | Included |
|  | Tooth mobility | 1. Have you ever had any teeth became loose on their own, without an injury? | Included |
|  | Bone loss | 1. Have you ever been told by a dental professional that you lost bone around your teeth? | Included |
|  | Tooth appearance | 1. During the past 3 months, have you noticed a tooth that doesn’t look right? | Included |
|  | Use of dental floss | 1. Aside from brushing your teeth with a toothbrush, in the last 7 days, how many times did you use dental floss or any other device to clean between your teeth? | Excluded (irrelevant to periodontal disease signs and symptoms) |
|  | Use of mouthwash | 1. Aside from brushing your teeth with a toothbrush, in the last 7 days, how many times did you use mouthwash or other dental rinse product that you use to treat dental disease or dental problems? | Excluded (irrelevant to periodontal disease signs and symptoms) |
| (Carra et al., 2018) | Gum bleeding | 1. Have your gums bled recently? | Included |
|  | Food Impaction | 1. Do you have food impaction between your teeth? | Excluded (irrelevant to periodontal disease signs and symptoms) |
|  | Gum recession | 1. Do you notice your teeth getting longer? | Included |
|  | Gum recession | 1. Do you think that you can see more roots of teeth than in the past? | Excluded to reduce redundancy |
| (Wright et al., 2021) | Gum problems | 1. I have gum problems | Excluded to reduce redundancy |
|  | Gingivitis diagnosis | 1. My doctor has told me I have gingivitis | Excluded to reduce redundancy |
|  | Mobility on Chewing | 1. I can feel my teeth move when I chew food | Included |
|  | Mobility | 1. I have teeth that seem loose or wiggly | Excluded to reduce redundancy |
|  | Gum Inflammation Diagnosis | 1. My dentist (or dental hygienist) said my gums are inflamed | Excluded to reduce redundancy |
|  | Foul breath | 1. My breath smells foul even after brushing | Excluded (Not specific to periodontitis) |
|  | Gum bleeding on brushing | 1. When I brush, my gums bleed | Excluded to reduce redundancy |
|  | Gum bleeding on flossing | 1. My gums bleed when I floss | Excluded to reduce redundancy |
|  | Gum recession | 1. My gums are receding | Excluded to reduce redundancy |
|  | Gum Swelling | 1. My gums feel swollen and puffy | Excluded (Not specific to periodontitis) |
|  | Missing teeth | 1. I have missing permanent teeth | Excluded to reduce redundancy |
|  | Difficulty in eating | 1. My gums make it difficult to eat | Excluded (Not specific to periodontitis and uncommonly reported by patients) |
|  | Difficulty in chewing | 1. I have difficulty chewing because of my teeth | Excluded (Not specific to periodontitis and uncommonly reported by patients) |
|  | Gum color | 1. I have really bright red gums | Excluded (Not specific to periodontitis) |
|  | Gum recession | 1. It seems like my gums are pulling away from my teeth | Excluded to reduce redundancy |
|  | Foul breath | 1. I have bad breath | Excluded (Not specific to periodontitis) |
|  | Gum tenderness | 1. I have tender gums | Included |
|  | Gum bleeding | 1. My gums often bleed | Excluded to reduce redundancy |
|  | Gum treatment | 1. I should get treatment for my gums | Excluded to reduce redundancy and irrelevant to periodontal disease signs and symptoms |
|  | Gum recession | 1. My teeth seem longer than they used to | Excluded to reduce redundancy |
|  | Metallic taste | 1. There is a constant metallic taste in my mouth | Excluded (Not commonly reported by patients) |
|  | Gum bleeding on eating hard food | 1. My gums bleed when I bite into a hard food such as an apple | Excluded (Not commonly reported by patients and to reduce redundancy) |
